# Influencing factors of physical exercise motivation among military academy cadets: a social ecological model analysis

**DOI:** 10.3389/fpsyt.2026.1741195

**Published:** 2026-03-03

**Authors:** Wenqi Wang, Chao Xu, Guoxian Gao, Cheng Zhang, Fengxiang Wang, Yanliang Song, Wei Wang

**Affiliations:** 1 Department of Military and Physical Education, Qingdao Branch of Naval Aviation University, Qingdao, China; 2 College of Physical Education, Guizhou University of Finance and Economics, Guiyang, China; 3 Department of Military Physical Education, Naval Aviation University, Yantai, China

**Keywords:** chain mediation, interpersonal support, military cadets, organizational support, physical exercise motivation, self-efficacy, social ecological model, structural equation modeling

## Abstract

**Background:**

Physical exercise motivation is a critical determinant of health behavior among military academy cadets. While individual psychological factors have been extensively studied, the multilevel influences based on the Social Ecological Model (SEM) remain underexplored in this population. This study aimed to investigate the effects of intrapersonal (exercise self-efficacy), interpersonal (social support), and Organizational (institutional support) factors on exercise motivation among Chinese military academy cadets.

**Methods:**

A validated questionnaire was developed based on SEM constructs. After pilot testing with 294 participants (Cronbach’s α ≥ 0.82 for all scales), a cross-sectional survey was conducted among 581 cadets. Structural Equation Modeling (SEM) was performed using AMOS 24.0 to examine direct and indirect pathways. Bootstrap resampling (5,000 iterations) was employed to test mediating effects.

**Results:**

Exercise self-efficacy demonstrated a significant total effect on exercise motivation (β = 0.465, *p* < 0.001, 95% CI [0.357–0.569]) but no significant direct effect, indicating full mediation. Both interpersonal support (β = 0.547, *p* < 0.001) and Organizational support (β = 0.198, *p* = 0.001) exhibited significant positive direct effects. Three significant indirect pathways were identified: (1) Self-efficacy → Organizational support → Motivation (β = 0.136, *p* = 0.005, 95% CI [0.038–0.268]). (2) Self-efficacy → Interpersonal support → Motivation (β = 0.130, *p* < 0.001, 95% CI [0.055–0.223]). (3) Self-efficacy → Organizational support → Interpersonal support → Motivation (β = 0.175, *p* < 0.001, 95% CI [0.115–0.255]).

**Conclusion:**

Organizational and interpersonal supports serve as critical mediators translating individual self-efficacy into exercise motivation. Multi-level interventions targeting policy optimization (e.g., mandatory group exercise programs), peer-mentoring systems, and instructor training may facilitate the transition from extrinsic compliance to intrinsic motivation among military cadets.

## 1 Introduction

Military academy cadets represent the future core of national defense capabilities. For this population, maintaining high levels of physical fitness and exercise motivation is not only a matter of personal health but a critical component of operational readiness and combat effectiveness (1). While extensive research has established the importance of exercise motivation in predicting physical activity adherence in general and student populations (2, 3), the unique institutional and social dynamics influencing motivation within military academies remain underexplored.

The Social Ecological Model (SEM) provides a robust framework for understanding health behaviors as outcomes of multi-level interactions (4). It posits that behavior is shaped by intrapersonal, interpersonal, Organizational, community, and policy factors. This model has been widely applied in civilian physical activity research (5). However, military academies constitute “total institutions” characterized by compulsory membership, hierarchical governance, physical seclusion, and a strong emphasis on collective norms (6). These features likely compress and intensify the influences of the Organizational and interpersonal layers of the SEM, while potentially altering the pathways through which individual factors, such as self-efficacy, operate. For instance, mandatory training regimens may shift the balance between intrinsic and extrinsic motivation, and peer influence may be amplified within closed, cohesive units. Despite the theoretical relevance of the SEM to this setting, few studies have empirically tested a multi-level SEM framework to explain exercise motivation specifically among military cadets.

To address this gap by applying a tailored SEM framework to the military academy context. We focus on three core, modifiable levels: (a) the intrapersonal level (exercise self-efficacy), (b) the interpersonal level (support from peers and instructors), and (c) the Organizational level (institutional support in terms of programs, facilities, and policy environment). By examining both the direct effects of these factors and their potential mediating relationships, this research seeks to elucidate the complex mechanisms driving exercise motivation in a high-stakes, structured environment. Clarifying these pathways is essential for developing ecologically valid interventions that can effectively promote self-determined, sustained physical training among future military officers, moving beyond mere compliance to fostering intrinsic engagement.

The innermost layer of the Social Ecological Model comprises individual characteristics. Sallis et al. (7) identified exercise self-efficacy (ESE) as the psychological construct most strongly associated with physical-activity behavior. Among adolescents and university students, ESE exerts a direct, positive effect on both intention and maintenance of exercise (8, 9). Because ESE represents the conviction that one can successfully execute regular physical activity, cadets with high ESE are more likely to experience positive affect during exercise, sustain pleasant affective states, and consequently adhere to training routines over time (10). Huang, M. R. et al. (11) further demonstrated that university students’ lifestyle physical activity is mediated by sport-related interest that is itself predicted by ESE. We therefore selected ESE as the key intrapersonal predictor.

Interpersonal support is typically supplied through family, peers, and teachers. In the cloistered context of military academies, cadets have minimal contact with parents; we therefore focus on peers and physical-education (PE) instructors. Zhang et al. (12) reported that peer and teacher support were the strongest social predictors of students’ self-reported exercise participation, second only to ESE. During adolescence, support from friends becomes the dominant social influence, exerting a decisive effect on both motivation and actual engagement (13). Teachers’ instructional style and professional competence are also significantly associated with students’ exercise behavior (14).

Organizational support encompasses school-level opportunities, community resources, and policy promotion. Given the sequestered nature of military academies, we concentrate on curricular provision, extracurricular programs, and access to sports facilities. Cross-sectional and observational studies consistently show that schools offering abundant, attractive physical-activity options foster more active lifestyles among students (15). Systematic observations further reveal that students’ activity levels vary with class size, teaching modality, and overall curricular climate (16, 17). Although policies are intended to promote activity, university students often perceive them as coercive and minimally motivating, sometimes eliciting reactance (18).

This study aimed to investigate the effects of intrapersonal (exercise self-efficacy), interpersonal (social support), and Organizational (institutional support) factors on exercise motivation among Chinese military academy cadets. Based on this objective, the present study proposes the following hypotheses:

H1. Exercise self-efficacy positively predicts military cadets’ exercise motivation.
H2. Organizational support positively predicts military cadets’ exercise motivation.
H3. Interpersonal support positively predicts military cadets’ exercise motivation.
H4. Organizational and interpersonal support serially mediate the relationship between exercise self-efficacy and exercise motivation.

## 2 Participants and methods

### 2.1 Sample and procedure

A cross-sectional survey design was employed. Participants were full-time undergraduate and junior-college cadets recruited from six Chinese military academies using a purposive sampling method to ensure diversity in training branches and geographic location. To mitigate potential coercion and respect the chain of command, the research team collaborated with academy administrative offices. Invitations containing a link/QR code to the anonymous online survey (hosted on Wenjuanxing platform) were disseminated through official cadet communication channels (e.g., intranet bulletins, class group chats), not directly by instructors to their own students, to minimize social desirability bias. Participation was voluntary, and informed consent was obtained electronically prior to survey commencement.

Data collection occurred in two phases: Pilot Study: An initial survey link was distributed in one academy. Out of 381 responses, 294 valid questionnaires were retained after applying validity checks (effective rate = 77.17%). Main Study: The revised survey was distributed across six academies. From 942 submitted questionnaires, 581 were deemed valid after screening (effective rate = 61.68%), comprising 315 undergraduates and 266 junior-college students. *Invalidity Criteria:* Questionnaires were excluded if they exhibited (a) straight-lining (identical responses to ≥ 10 consecutive items), (b) implausibly short completion time (< 1/3 of median time), or (c) more than 10% of items left blank.

### 2.2 Instrument

We developed the Military Cadet Exercise-Motivation Determinants Scale (MC-EMDS) through a multi-step, rigorous process to ensure its validity and reliability for the target population.

Item Generation and Expert Review: An initial item pool of 59 statements was generated based on a comprehensive literature review of SEM constructs in physical activity (e.g., 12, 19) and semi-structured interviews with 15 cadets from two academies (not involved in the main study) to capture context-specific nuances. The items were designed to measure four first-order latent constructs: Exercise Self-Efficacy, Interpersonal Support, Organizational Support, and Exercise Motivation, with further delineation into six second-order facets (20–23) (see **Table 1** for sources).

**Table 1:** Scale dimensions and source items.

1st-order factor	2nd-order factor	Item numbers	Source
Individual factors	Exercise self-efficacy	Q1, Q5, Q7, Q11, Q15, Q20, Q23, Q25, Q32	Lee, L. L., Chiu, Y. Y., Ho, C. C., et al. (2011). The Chinese version of the Outcome Expectations for Exercise scale: Validation study. *International Journal of Nursing Studies*, 48(6), 672-680.
Inter-personal support	Peer support	Q21, Q27, Q34, Q45	Guo, K. L. (2019). The relationship among school sports environment, exercise intention and physical activity in junior-high students (Master’s thesis, Shanghai University of Sport).
	Teacher support	Q18, Q31, Q33, Q37, Q48	Chen, P. Y., & Sun, Q. Z. (2014). Construction of a social-ecological model for promoting youth physical activity—Evidence from primary and secondary students in Jiangsu. *Journal of Shanghai University of Sport*, 38(5), 79-84.
Organizational support	Curriculum & activities	Q14, Q16, Q29, Q42, Q44	Guo, K. L. (2019). The relationship among school sports environment, exercise intention and physical activity in junior-high students (Master’s thesis, Shanghai University of Sport).
	Facilities	Q9, Q17, Q35, Q46	Huang, M. R., & Zhang, Y. P. (2020). A social-ecological reflection on factors influencing university students’ sport-life style in China. *Sports & Science*, 41(3), 110-120.
	Policy & publicity	Q3, Q28, Q40, Q41, Q43, Q47	—
Exercise motivation	—	Q2, Q4, Q6, Q8, Q10, Q12, Q13, Q19, Q22, Q24, Q26, Q30, Q36, Q38, Q39	Chen, S. P., Wang, Y. B., & Rong, J. Z. (2013). Development and validation of a simplified Motives for Physical Activity Measure-Revised (MPAM-R) for Chinese exercisers. *Journal of Beijing Sport University*, 36(2), 66-70 + 78.

To ensure content validity and relevance to the military context, a panel of seven experts was convened. The panel included three associate professors specializing in military physical education, two sports psychologists, one senior military training officer, and one methodological with expertise in scale development. Experts were selected based on their publication record in related fields and practical experience with the cadet population. They independently rated each item on a 4-point scale for relevance, clarity, and comprehensiveness. The Scale-Level Content Validity Index (S-CVI/Average) was calculated as 0.97, and the Item-Level CVI (I-CVI) ranged from 0.86 to 1.00, exceeding the recommended threshold of 0.78 (24). Based on expert feedback regarding redundancy and clarity, the item pool was refined to 48 items. Importantly, the expert panel also assessed the theoretical alignment of the item structure with the SEM framework, confirming its appropriateness.

Pilot Testing and Scale Refinement: The 48-item scale was administered in the pilot study (N = 294). We acknowledge the limitation noted by Reviewer 1 regarding the absence of test-retest reliability in the pilot phase. While internal consistency was high (Cronbach’s α ≥ 0.82 for all major constructs), future iterations of the scale should include temporal stability assessment. Exploratory Factor Analysis (EFA) was conducted using principal axis factoring with Promax rotation (allowing for correlated factors as theorized in SEM). The Kaiser-Meyer-Olkin measure was 0.938, and Bartlett’s test was significant (p <.001). Items were retained if they met the following criteria: (1) primary factor loading ≥ 0.50, (2) cross-loading difference ≥ 0.20, and (3) theoretical coherence with the intended construct. This process led to the deletion of 8 items, resulting in a final 40-item scale with a clear four-factor structure (**Table 2**), accounting for 60.95% of the total variance. All retained factors demonstrated acceptable internal consistency (Cronbach’s α > 0.70).

**Table 2:** Factor loadings and factor labels.

Factor	Item (English translation)	Loading	Variance explained
Inter-personal support	Q34. I am more willing to exercise when friends take part.	.836	19.82%
	Q45. My friends often encourage me to exercise.	.745	
	Q31. My PE instructor is conscientious and responsible.	.742	
	Q48. My squad officers strongly support my exercise participation.	.716	
	Q37. My squad officers praise students who excel in both academics and sport equally.	.704	
	Q27. My friends often exercise together with me.	.638	
Exercise self-efficacy	Q23. I am confident I can exercise even when I feel depressed.	.775	16.77%
	Q15. I am confident I can exercise even when I feel pain.	.724	
	Q7. I am confident I can exercise even when I feel tired.	.696	
	Q25. I am confident I can exercise even when the weather bothers me.	.693	
	Q32. I am confident I can exercise even when I am busy.	.692	
	Q20. I am confident I can exercise even when I am not interested in the activity.	.686	
Exercise motivation	Q4. I exercise to control my weight.	.787	13.12%
	Q13. I exercise to keep or improve my figure.	.753	
	Q12. I exercise to make myself more attractive.	.691	
	Q6. I exercise to improve my sport skills.	.582	
Organizational support	Q17. I often use academy facilities for sports organized by my university/college/unit.	.868	11.24%
	Q16. I often take part in sports activities organized by my university/college/unit.	.812	
	Q3. Campus publicity (honor boards, slogans) motivates me to exercise.	.480	
	Q14. My university/college/unit regularly holds sports events (sports meets, basketball games, fitness contests, etc.).	.469	

Final Scale Structure: The final MC-EMDS comprised 40 items rated on a 5-point Likert scale (1=Strongly Disagree to 5=Strongly Agree). The four latent constructs were measured as follows:

(1) Exercise Self-Efficacy (ESE): 6 items (e.g., “I am confident I can exercise even when I feel tired”). (2) Organizational Support (OS): 4 items covering curriculum/activities and facilities (e.g., “I often use academy facilities for sports organized by my unit”). (3) Interpersonal Support (IS): 6 items covering peer and teacher/squad leader support (e.g., “My friends often encourage me to exercise”). (4) Exercise Motivation (MOT): 4 items focusing on intrinsic and identified regulation aspects (e.g., “I exercise to improve my sport skills”).

Content validity was evaluated by seven additional subject-matter experts using a 4-point relevance scale (≥ 3 = relevant). S-CVI (scale-level content-validity index, average method) was 0.97. All first-order factors achieved S-CVI ≥ 0.93; second-order factors ranged from 0.86 (Facilities) to 1.00 (Exercise Self-Efficacy, Curriculum/Activities).

### 2.3 Data analysis

#### 2.3.1 Data were analyzed using SPSS 26.0 and AMOS 26.0.

##### 2.3.1.1 Confirmatory factor analysis

A CFA was performed on the main study sample (N = 581) to validate the four-factor structure derived from the EFA. Model fit was assessed using multiple indices: χ²/df (<5), Comparative Fit Index (CFI >0.90), Tucker-Lewis Index (TLI >0.90), Root Mean Square Error of Approximation (RMSEA <0.08), and Standardized Root Mean Square Residual (SRMR <0.08) (25). Convergent validity was assessed via Average Variance Extracted (AVE >0.50) and Composite Reliability (CR >0.70). Discriminant validity was confirmed if the square root of the AVE for each construct was greater than its correlations with other constructs (26).

##### 2.3.1.2 Common method bias assessment

Given the cross-sectional, self-report design, we employed both procedural and statistical controls. Procedurally, we ensured respondent anonymity and item randomization. Statistically, we used the latent common method factor approach (27). A method factor linking all items was added to the measurement model. A significant improvement in model fit (ΔCFI/ΔTLI > 0.10, ΔRMSEA > 0.05) would indicate substantial CMB. As shown in Results, the change was negligible.

##### 2.3.1.3 Structural equation modeling

The hypothesized structural model was tested to examine direct paths from ESE, OS, and IS to MOT, as well as the indirect paths specified in H4.

##### 2.3.1.4 Mediation analysis

The significance of indirect effects was tested using bias-corrected bootstrap confidence intervals with 5,000 resamples. An indirect effect is considered statistically significant if its 95% CI does not include zero. We emphasize that the cross-sectional design precludes definitive causal claims; the terms “effect” and “mediation” are used within the statistical modeling context to describe associations and indirect pathways.

## 3 Results

### 3.1 Pilot survey

#### 3.1.1 Item analysis

Critical-ratio (CR) testing was used. Total scores were ranked; the top and bottom 27% were labelled high (code 2) and low (code 1) groups. Independent-samples t-tests (SPSS 23.0) showed all p (two-tailed) <.001, indicating satisfactory discrimination; no items were deleted.

#### 3.1.2 Reliability

Cronbach’s α for the pilot sample (N = 294) was.956 overall. First-order factors: Individual.849, Interpersonal Support.892, Organizational Support.892, Exercise Motivation.907. Second-order factors: Exercise Self-Efficacy.849, Peer Support.839, Teacher Support.797, Curriculum/Activities.793, Facilities.606, Policy/Promotion.824. All coefficients exceeded the.60 minimum, demonstrating acceptable internal consistency (28).

#### 3.1.3 Exploratory factor analysis

KMO = .938, Bartlett χ² p <.001, indicating suitability for factor extraction. Principal-axis factoring with varimax rotation yielded four factors (eigen-values > 1) explaining 60.95% of the variance after deleting items loading <.45 or cross-loading ≥.40. Factor loadings ranged.469–.868 (**Table 2**).

### 3.2 Results of confirmatory factor analysis

#### 3.2.1 Structural validity

**Figure 1:**
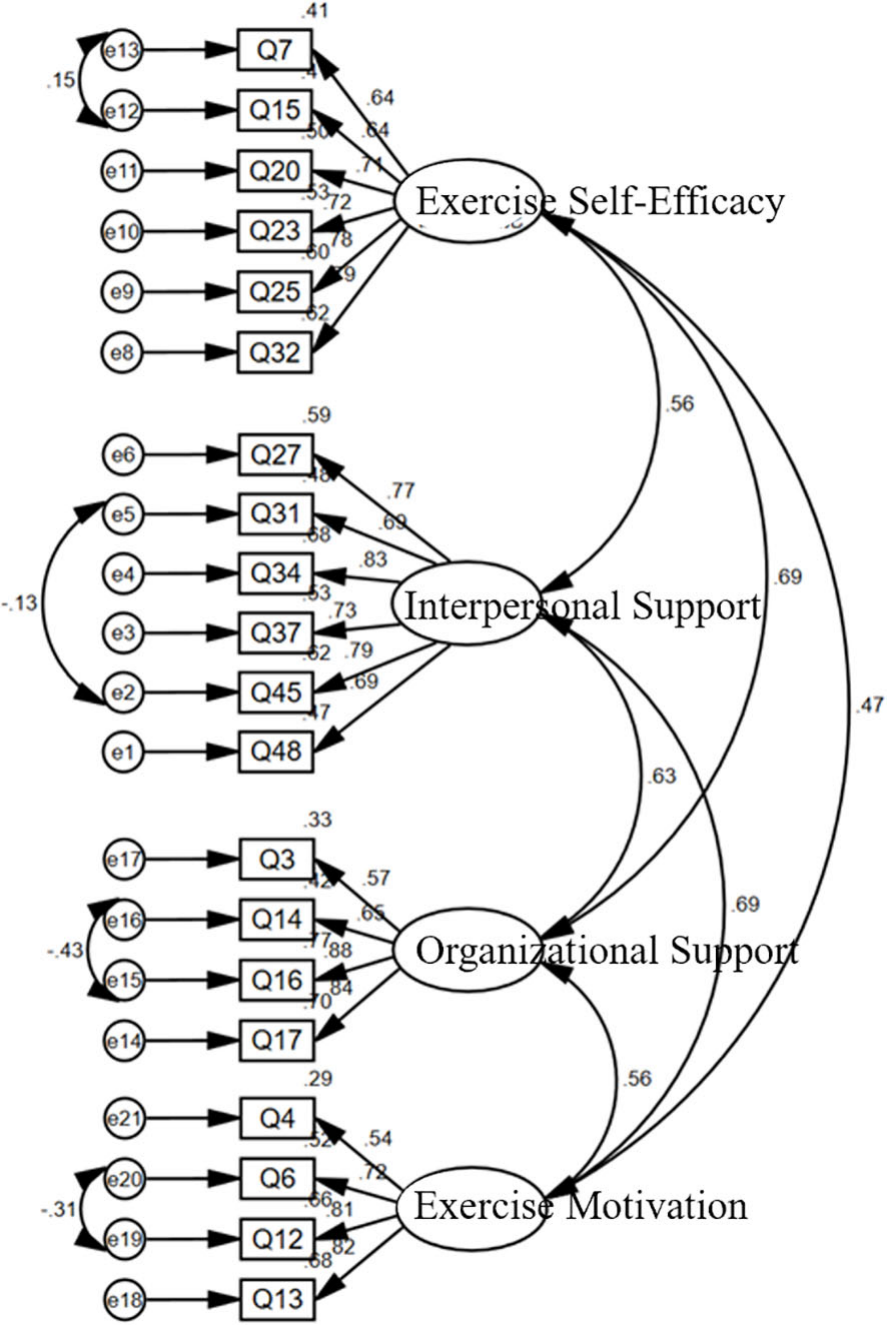
Path diagram of the confirmatory factor analysis model.

A confirmatory factor analysis (CFA) was conducted on the four latent constructs—exercise self-efficacy, interpersonal support, Organizational support, and exercise motivation. The path diagram is shown in **Figure 1**, and the model-fit indices are reported in **Tables 3**–**5** .

**Table 3:** Model fit indices for CFA.

	χ²/df	RMSEA	GFI	TLI	CFI	PNFI	PCFI
Criterion	1–5	<.08	>.90	>.90	>.90	>.50	>.50
Obtained value	3.251	.062	.917	.926	.938	.769	.790

**Table 4:** Composite reliability and convergent validity.

Factor	Item	STD.	SMC	CR	AVE
Exercise Self-Efficacy	Q32	0.785	0.617	0.862	0.511
	Q25	0.778	0.605		
	Q23	0.725	0.525		
	Q20	0.706	0.499		
	Q15	0.641	0.410		
	Q7	0.640	0.410		
Organizational Support	Q17	0.837	0.700	0.830	0.557
	Q16	0.880	0.774		
	Q14	0.649	0.422		
	Q3	0.575	0.330		
Exercise Motivation	Q13	0.822	0.675	0.819	0.537
	Q12	0.814	0.662		
	Q6	0.719	0.517		
	Q4	0.541	0.292		
Inter-personal Support	Q48	0.689	0.475	0.885	0.562
	Q45	0.786	0.618		
	Q37	0.731	0.535		
	Q34	0.826	0.682		
	Q31	0.691	0.477		
	Q27	0.767	0.588		

Note: Bold values represent Composite Reliability (CR) and Average Variance Extracted (AVE) for each factor. As shown in **Table 4**, the standardized factor loadings are distributed in a gradient fashion: items Q3 and Q4 fall in the 0.50–0.60 range, Q7, Q14, Q15, Q31, and Q48 lie between 0.60 and 0.70, while all remaining items exceed 0.70, indicating a generally high representativeness of the indicators for their underlying constructs. Moreover, composite reliability (CR) for each factor is above 0.80 and average variance extracted (AVE) exceeds 0.50, demonstrating satisfactory convergent validity and providing a solid foundation for the subsequent structural model analysis.

**Table 5:** Correlations and discriminant validity.

Factor	AVE	Inter-personal support	Exercise motivation	Organizational support	Exercise self-efficacy
Inter-personal Support	0.562	**0.749**			
Exercise Motivation	0.537	0.685	**0.733**		
Organizational Support	0.557	0.629	0.559	**0.746**	
Exercise Self-Efficacy	0.511	0.557	0.465	0.686	**0.715**

Bold figures on the diagonal are the square roots of AVE.

Since these square-root values exceed the correlations below them, discriminant validity among the four dimensions is confirmed.

As shown in **Table 3**, the χ²/df value is 3.251 (< 5), RMSEA = 0.062 (< 0.08), CFI = 0.938 (> 0.90), TLI = 0.926 (> 0.90), GFI = 0.917 (> 0.90), PNFI = 0.769 (> 0.50), and PCFI = 0.790 (> 0.50). All indices meet conventional criteria, indicating satisfactory structural validity. Because good structural validity is a prerequisite for convergent and discriminant validity (29), composite reliability and convergent validity were subsequently computed from the CFA results.

#### 3.2.2 Composite reliability and convergent validity

### 3.3 Multicollinearity test

To address potential concerns regarding multicollinearity among the predictor variables (Exercise Self-Efficacy, Organizational Support, and Interpersonal Support), collinearity diagnostics were conducted. As shown in **Table 6**, the Variance Inflation Factor (VIF) values for all constructs ranged from 1.740 to 2.267, which are well below the conservative threshold of 5. Correspondingly, tolerance values exceeded 0.1. These results confirm that multicollinearity is not a significant issue in the present data.

**Table 6:** Collinearity diagnostics for predictor variables.

Variable (latent construct)	Tolerance	VIF
Exercise Self-Efficacy	0.503	1.987
Organizational Support	0.441	2.267
Interpersonal Support	0.575	1.740

### 3.4 Common-method bias test

**Figure 2:**
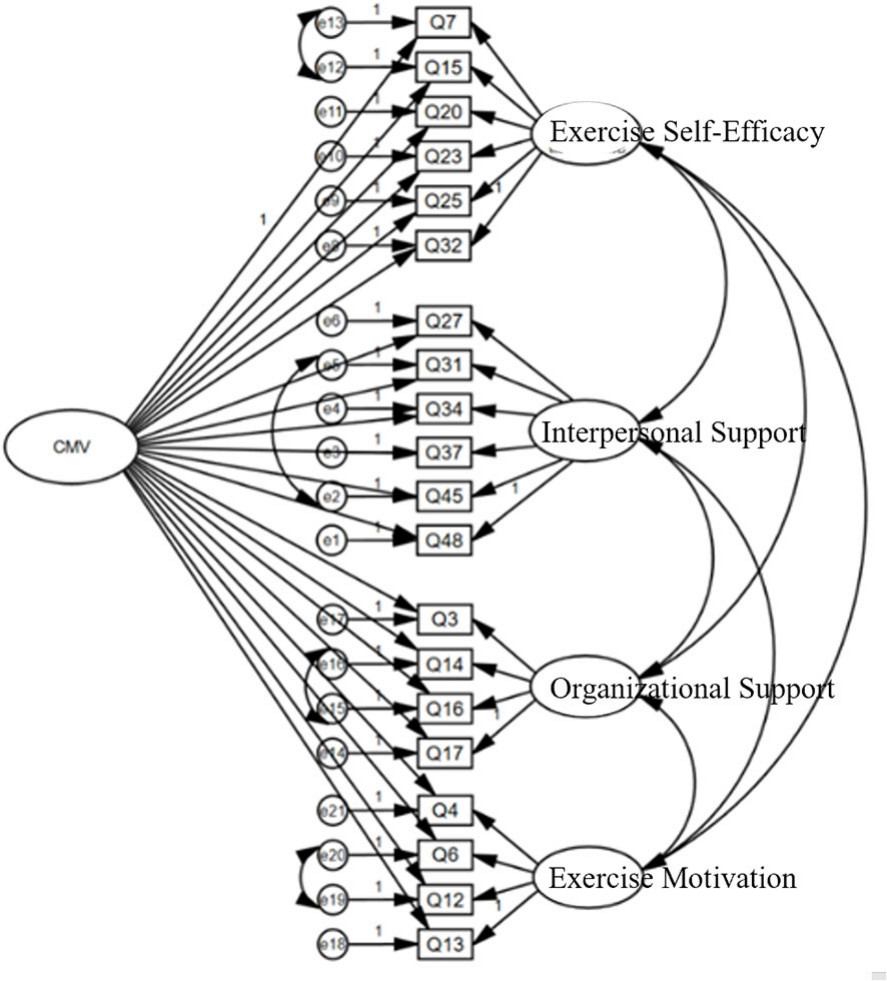
Bifactor CFA model.

**Figure 3:**
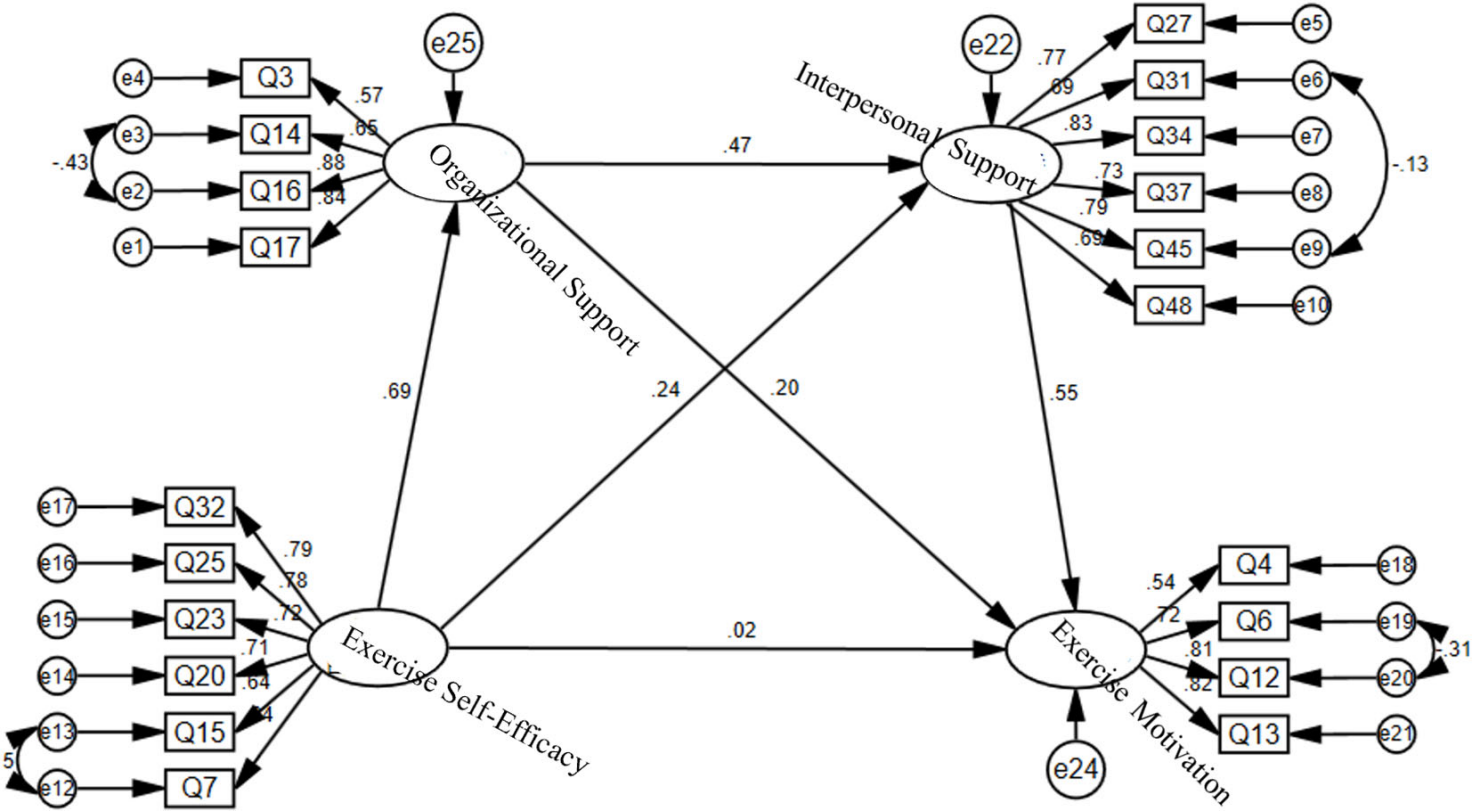
Path analysis of the structural equation model.

We used the latent-factor control approach to assess potential common-method bias. A global method factor was added to the original CFA model, yielding a bifactor specification. As shown in **Table 6**, **Figure 2**, the incremental fit after introducing the method factor was:Δχ²/df = 1.024, ΔCFI = 0.032, ΔTLI = 0.034, ΔRMSEA = –0.016, and ΔSRMR = –0.031. Because the increases in CFI and TLI were < 0.10 and the decreases in RMSEA and SRMR were < 0.05, the improvement in fit is trivial (30), indicating that common-method bias is not a serious threat in the present data (**Figure 3**).

### 3.5 Robustness check: competitive model analysis

To verify the robustness of the hypothesized serial mediation model, a competitive model analysis was conducted. We compared the hypothesized model with an alternative parallel mediation model, in which the direct path from Organizational support to interpersonal support was removed.

As shown in **Table 6**, the fit indices for the competitive model (χ2 = 580.654, CFI = 0.928, TLI = 0.915, RMSEA = 0.067) were inferior to those of the hypothesized model (χ2 = 520.099, CFI = 0.938, TLI = 0.926, RMSEA = 0.062). Furthermore, the Information Criteria for the hypothesized model (AIC = 620.099, BIC = 838.336) were substantially lower than those for the competitive model (AIC = 678.654, BIC = 892.527), indicating a superior trade-off between model fit and parsimony. These results support the validity of the serial mediation structure over a parallel alternative.

### 3.6 Hypothesis testing

#### 3.6.1 Path test

As shown in **Table 7**, **Figure 2**, after controlling for the mediating effects, the direct-path tests among variables in the hypothesized model yielded the following results: exercise self-efficacy exerted a significant positive effect on Organizational support (C.R. = 12.369, p < 0.001) and on interpersonal support (C.R. = 3.981, p < 0.001), but its influence on exercise motivation was non-significant (C.R. = 0.434, p = 0.664); Organizational support significantly and positively predicted both interpersonal support (C.R. = 7.793, p < 0.001) and exercise motivation (C.R. = 3.176, p = 0.001), thereby supporting Hypothesis 2 that Organizational support is positively related to military cadets’ exercise motivation; interpersonal support also had a significant positive effect on exercise motivation (C.R. = 8.284, p < 0.001), supporting Hypothesis 3 that interpersonal support is positively related to military cadets’ exercise motivation. **Tables 8**–**10**.

**Table 7:** Comparison of model fit indices.

Model	X²/df	CFI	TLI	RMSEA	SRMR
Without method factor	3.251	0.938	0.926	0.062	0.062
With method factor	2.227	0.970	0.960	0.046	0.031
Difference (Δ)	1.024	0.032	0.034	0.016	0.031

**Table 8:** Comparison of fit indices between the hypothesized and competitive models.

Model	χ2	df	CFI	TLI	RMSEA	AIC	BIC
Hypothesized Model (Serial Mediation)	520.099	160	0.938	0.926	0.062	620.099	838.336
Competitive Model(Parallel Mediation)	580.654	161	0.928	0.915	0.067	678.654	892.527

**Table 9:** Path analysis of the hypothesized model.

Path	UNSTD	S.E.	C.R.	P	STD	Hypothesis
Organizational Support ← Exercise Self-Efficacy	0.813	0.066	12.369	***	0.686	Supported
Interpersonal Support ← Organizational Support	0.335	0.043	7.793	***	0.466	Supported
Interpersonal Support ← Exercise Self-Efficacy	0.202	0.051	3.981	***	0.237	Supported
Exercise Motivation ← Organizational Support	0.114	0.036	3.176	0.001	0.198	Supported
Exercise Motivation ← Exercise Self-Efficacy	0.017	0.039	0.434	0.664	0.025	Not supported
Exercise Motivation ← Interpersonal Support	0.441	0.053	8.284	***	0.547	Supported

***Indicates statistical significance at p < 0.001; S.E., standard error; C.R., critical ratio.

**Table 10:** Bootstrap test for multiple mediation.

Path	Effect	STD	SE	P	95%CL	
					LB	UB
Exercise self-efficacy → Exercise motivation	Total effect	0.465	0.054	0.000	0.357	0.569
	Direct effect	0.025	0.066	0.724	-0.106	0.151
	ESE → Organizational support → Motivation	0.136	0.057	0.005	0.039	0.268
	ESE → Interpersonal support → Motivation	0.130	0.043	0.000	0.055	0.223
	ESE → Organizational support → Interpersonal support → Motivation	0.175	0.035	0.000	0.115	0.255
	Total indirect effect	0.440	0.062	0.000	0.357	0.569

#### 3.6.2 Mediation test

A bootstrap test based on 5,000 resamples showed that the total effect of exercise self-efficacy on exercise motivation was 0.465 (SE = 0.054, p < 0.001) with a 95% confidence interval of [0.357, 0.569] that did not include zero, so the total effect was significant and Hypothesis 1 was supported. After controlling for the mediators, the direct effect was 0.025 (SE = 0.066, p = 0.724) with a 95% confidence interval of [–0.106, 0.151] that included zero, indicating a non-significant direct effect.

Among the multiple mediation paths, the effect of exercise self-efficacy → Organizational support → exercise motivation was 0.136 (SE = 0.057, p = 0.005, 95% CI [0.039, 0.268]), the effect of exercise self-efficacy → interpersonal support → exercise motivation was 0.130 (SE = 0.043, p < 0.001, 95% CI [0.055, 0.223]), and the effect of exercise self-efficacy → Organizational support → interpersonal support → exercise motivation was 0.175 (SE = 0.035, p < 0.001, 95% CI [0.115, 0.255]), none of whose confidence intervals included zero, so all indirect effects were significant, supporting Hypothesis H4 that Organizational and interpersonal support serially mediate the relationship between exercise self-efficacy and exercise motivation; the total indirect effect was also significant at 0.440 (SE = 0.062, p < 0.001, 95% CI [0.357, 0.569]).

In sum, exercise self-efficacy has no direct effect on exercise motivation and its influence is fully mediated through the three indirect paths, confirming the existence of a multiple-mediation mechanism.

## 4 Discussion

This study applied a Social Ecological Model framework to investigate multi-level factors influencing exercise motivation among Chinese military academy cadets. The key finding is that exercise self-efficacy influences motivation indirectly and fully, through its positive associations with perceived Organizational and interpersonal support, rather than through a direct pathway. This pattern of full mediation, coupled with the strong direct effects of support systems, highlights the paramount importance of the institutional and social environment in shaping motivational outcomes within this unique, structured setting.

Our finding that self-efficacy had no direct effect on motivation contrasts with numerous studies in civilian university settings where a direct, strong link is often reported (e.g., 29, 31). This discrepancy is theoretically significant and likely reflects the core distinction of the military academy environment. In a context where physical training is largely mandatory, standardized, and oriented towards collective performance and assessment, an individual’s confidence in their capability (self-efficacy) may not freely translate into autonomous motivation (32). Instead, the behavior is often externally regulated. High self-efficacy in this setting might primarily enhance a cadet’s perception of and engagement with the provided resources and social networks (i.e., “I am confident, so I am more likely to utilize facilities and seek out peers”), which then become the proximal drivers of motivation. This aligns with Huang and Zhang (18), who suggested that in highly regulated environments, the “conversion efficiency” of self-efficacy into intrinsic motivation can be attenuated. Our results empirically demonstrate this contextual effect, showing that self-efficacy’s role is to activate and amplify the benefits of the environmental supports, rather than to directly fuel motivation independently.

Regarding the Organizational level, our findings revealed that Organizational support had a significant, though moderate, direct effect on motivation. The items with the highest loadings pertained to accessible facilities and organized activities, whereas policy/publicity items were weaker. This suggests that for cadets, tangible, actionable resources are more motivating than top-down promotional messages. Well-maintained gyms, regular intra-unit competitions, and diverse training programs provide the essential “opportunity structure” that makes exercise feasible and engaging (19). In the military context, these Organizational provisions also carry normative weight, signaling institutional priority on physical fitness, which can legitimize and valorize exercise participation.

At the interpersonal level, the results indicated that interpersonal support emerged as the strongest direct predictor of motivation. This underscores the critical role of the immediate social milieu within the “total institution.” Peer support, characterized by companionship and shared participation, likely fosters a sense of belonging and turns exercise into a social ritual, increasing adherence through social obligation and enjoyment (13). Support from instructors and squad leaders, which includes professional guidance and recognition, satisfies the psychological need for competence and can help internalize the value of training (33). In a closed community where peers and superiors are constant presences, their attitudes and behaviors become powerful immediate influences, potentially outweighing the impact of broader Organizational policies.

Furthermore, the mediation analysis highlighted that the most substantial indirect effect flowed through the serial pathway (ESE→OS→IS→MOT). This reveals a potential mechanism: cadets with higher self-efficacy are more likely to perceive and take advantage of Organizational resources (e.g., use the gym, join a team). This active engagement, in turn, places them in social interactions where they receive encouragement and recognition from peers and leaders, which ultimately strengthens their motivation. This chain highlights the synergistic interplay between environmental affordances and social processes. It suggests that interventions solely improving facilities may have limited impact unless they also create opportunities for positive social interaction around those facilities.

Despite its contributions, this study has several limitations that must be acknowledged, as rightly pointed out by the reviewers. First, the cross-sectional design limits causal inference. Longitudinal or experimental studies are needed to confirm the directionality of the proposed pathways. Second, reliance on self-reported data may introduce bias, though we attempted to mitigate this through anonymity and CMB assessment. Future research could incorporate objective measures of physical activity or peer-network analysis. Third, the sample was drawn from Chinese military academies, which may limit the generalizability of findings to other cultural or military contexts. Comparative studies across different nations’ military training systems would be valuable. Fourth, while our scale demonstrated good psychometric properties, the absence of test-retest reliability data and the use of a relatively homogeneous expert panel for content validation are methodological constraints for future work to address.

## 5 Conclusions and recommendations

From a theoretical perspective this study contributes to the literature by empirically validating a multi-level Social Ecological Model within the under-researched context of military academies. It demonstrates that in highly structured, collective environments, the pathway from individual psychological assets (self-efficacy) to behavioral motivation is not direct but is fully channeled through the perceived environment (Organizational and interpersonal support). This finding calls for a nuanced application of general psychological theories to specific institutional contexts.

In terms of practical implications the results suggest that to enhance cadets’ exercise motivation, multi-faceted interventions are necessary: (1) Strengthen Organizational Support: Beyond mandatory training, diversify physical activity offerings (e.g., elective sports, adventure training) and ensure easy access to high-quality, well-maintained facilities. Policy should focus on enabling and enriching the exercise experience rather than merely mandating it. (2) Leverage Interpersonal Dynamics: Foster a positive motivational climate by training instructors and squad leaders to provide autonomy-supportive feedback and recognize effort. Create structured peer-mentoring systems or small-group training challenges to capitalize on peer influence. (3) Create Synergistic Interventions: Design programs that combine resource provision with social interaction. For example, new facility openings could be paired with instructor-led workshops or inter-unit tournaments, facilitating the serial pathway from resource use to social support and enhanced motivation.

By adopting such an ecologically informed approach, military academies can more effectively cultivate not only physically fit, but also intrinsically motivated, future officers.

## Data Availability

The original contributions presented in the study are included in the article/supplementary material. Further inquiries can be directed to the corresponding author/s.
